# Comparison of Replication-Competent, First Generation, and Helper-Dependent Adenoviral Vaccines

**DOI:** 10.1371/journal.pone.0005059

**Published:** 2009-03-31

**Authors:** Eric A. Weaver, Pramod N. Nehete, Stephanie S. Buchl, Julien S. Senac, Donna Palmer, Philip Ng, K. Jagannadha Sastry, Michael A. Barry

**Affiliations:** 1 Department of Internal Medicine, Division of Infectious Diseases, Translational Immunovirology Program, Mayo Clinic, Rochester, Minnesota, United States of America; 2 Department of Immunology, Mayo Clinic, Rochester, Minnesota, United States of America; 3 Department of Molecular Medicine, Mayo Clinic, Rochester, Minnesota, United States of America; 4 Department of Veterinary Sciences, The University of Texas M.D. Anderson Cancer Center, Houston, Texas, United States of America; 5 Department of Molecular and Human Genetics, Baylor College of Medicine, Houston, Texas, United States of America; 6 Department of Immunology, The University of Texas M.D. Anderson Cancer Center, Houston, Texas, United States of America; Institut Pasteur Korea, Republic of Korea

## Abstract

All studies using human serotype 5 Adenovirus (Ad) vectors must address two major obstacles: safety and the presence of pre-existing neutralizing antibodies. Helper-Dependent (HD) Ads have been proposed as alternative vectors for gene therapy and vaccine development because they have an improved safety profile. To evaluate the potential of HD-Ad vaccines, we compared replication-competent (RC), first-generation (FG) and HD vectors for their ability to induce immune responses in mice. We show that RC-Ad5 and HD-Ad5 vectors generate stronger immune responses than FG-Ad5 vectors. HD-Ad5 vectors gave lower side effects than RC or FG-Ad, producing lower levels of tissue damage and anti-Ad T cell responses. Also, HD vectors have the benefit of being packaged by all subgroup C serotype helper viruses. We found that HD serotypes 1, 2, 5, and 6 induce anti-HIV responses equivalently. By using these HD serotypes in heterologous succession we showed that HD vectors can be used to significantly boost anti-HIV immune responses in mice and in FG-Ad5-immune macaques. Since HD vectors have been show to have an increased safety profile, do not possess any Ad genes, can be packaged by multiple serotype helper viruses, and elicit strong anti-HIV immune responses, they warrant further investigation as alternatives to FG vectors as gene-based vaccines.

## Introduction

A multitude of viral and non-viral vectors are being developed as vaccines for HIV-1. Adenoviral (Ad) vectors are arguably one of the most potent gene delivery and vaccine vectors available [1], [2], [3], [4], [5], [6]. The vast majority of gene therapy and vaccine studies have been performed using human serotype 5 Ad (Ad5). While Ad vectors are robust gene delivery vehicles, they are also very immunogenic [7].

**Figure 1:**
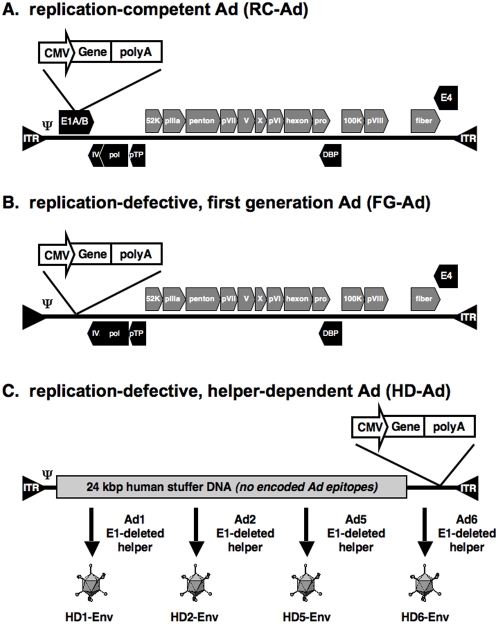
Schematic representation of adenovirus genome organization for replication-competent (A), First-Generation (B), and Helper-Dependent (C) viruses.

Innate and adaptive immune responses induced by first-generation adenovirus (FG-Ad) present several obstacles. First, innate immune responses induced by intravenous injection of FG-Ad results in the release of massive amounts inflammatory cytokines, such as IL-6 and TNF-α, within 3 to 24 hours [8], [9]. The events produced by intravenous administration of large doses can also lead to lethal events [10], [11]. Second, most work with Ad vectors utilizes FG-Ad vectors that are replication-defective due to a deletion of the E1 gene (Fig. 1B). While they are replication-defective, these vectors still carry most of the other Ad genes. These Ad genes can be expressed in transduced cells and be presented by MHC I and MHC II molecules to immune effector cells. Because of this, cytotoxic T lymphocytes (CTLs) can recognize Ad proteins in transduced cells and eliminate these cells within two to three weeks after vector administration [12], [13]. Finally, neutralizing antibodies are also a significant obstacle for the use of Ad5 viral vectored vaccines. As much as 27.3 to 50% of humans have pre-existing neutralizing antibodies against Ad5 [14]. In addition, antibodies are generated with each administration of Ad vector. These neutralizing antibodies can bind, inactivate and attenuate subsequent gene delivery by these viral vectors (10).

FG-Ads were created as a safer vector platform with increased transgene capacity than replication-competent Ad (RC-Ad) vectors. Helper-dependent Ad (HD-Ad) vectors were produced to further increase the safety and cloning capacity of Ad vectors. In HD-Ad vectors, all viral genes are deleted eliminating expression of potentially toxic and immunogenic viral proteins in transduced cells (Fig. 1C). For this reason, HD-Ads generate markedly reduced immune responses against themselves and their transgene proteins [15], [16], [17], [18]. This reduced immunogenicity allows for transgene expression in mice and in baboons in some cases over years [10], [19], [20], [21], [22]. This also allows HD-Ad vectors to produce significantly lower liver damage after i.v. injection than FG-Ad vectors [23]. For these reasons, HD-Ad vectors are well recognized in gene therapy applications to have improved safety. More recently HD-Ad has been explored as a platform to generate immune responses against transgene products like β-galactosidase [24]. In this case, HD-Ad vectors generated stronger T cell and antibody responses than FG-Ad in mice suggesting they may have utility as vaccine vectors.

The HD-Ad system is also particularly well suited to serotype switching, since adenoviruses in the same subgroup can generally cross-package each other's genomes. For example, an HD-Ad vector bearing a packaging signal and ITRs from subgroup C Ad5 can be cross-packaged by subgroup C Ad2 [21], [25]. This allows one to evade neutralizing antibodies by “serotype switching” the vector with different capsid antigens. For example, mice immunized with Ad2 serotype vectors generate potent neutralizing antibodies against Ad2 that drastically reduce transgene expression if Ad2 is used again [25]. However, if an Ad2 vector is used for first injection and an Ad5 vector is used for a second injection, there is little reduction in transduction because the Ad2-specific antibodies do not overtly neutralize the different Ad5 serotype [25]. Similarly, in baboons, serotype switching between Ad2 and Ad5 vectors allowed repeat administration in the face of neutralizing antibodies generated by the first vector [21], [26]. This approach has been applied more recently for FG-Ad HIV vaccines and has demonstrated the ability to markedly enhance vaccine responses [27], [28], [29].

Given their lower immunogenicity, increased safety, and recent problems related to vector-specific immune responses against Ad vaccines, this manuscript explores the utility of HD-Ad vectors as vaccines against HIV-1. In this work, we first compared the *in vivo* expression and vector-specific and transgene-specific immune responses generated by replication-competent Ad (RC-Ad), FG-Ad, and HD-Ad, all expressing the same transgene. We then compared the ability of HD-Ad and FG-Ad to drive immune responses against the HIV-1 envelope antigen. We also tested the utility of serotype-switching one Ad5 HD-Ad vector with subgroup C Ad1, Ad2, and Ad6 in mice and in rhesus macaques. This study is the first example of direct comparison of *in vivo* gene delivery by imaging and vector and transgene-specific immune responses driven by RC-Ad, FG-Ad, and HD-Ad vaccines. This is also the first study to investigate HD-Ad vectors and multiple serotype-switching for use as a HIV-1 vaccine in mouse and non-human primate models.

## Materials and Methods

### Adenoviruses

First generation replication defective (E1/E3 deleted) Ad5 vectors expressing the green-fluorescent protein-luciferase fusion protein GFPLuc and expressing HIV-1 Env gp140 from the subtype B strain JRFL were produced by the Ad-Easy system in 293A cells. Replication-competent Ad5 expressing GFPLuc was generated by insertion of the CMV-GFPLuc cassette between E1A and B as described in [30]. HD-Ad viruses expressing GFPLuc and Env were produced as previously described [31]. Briefly, the CMV-transgene-SV40 poly A cassettes from the FG-Ad vectors were PCR amplified, cloned, sequenced, and ligated into the Asc I site in the HD-Ad vector pΔ28-E4 (Fig. 1) [32]. Each HD-Ad plasmid backbone was cut with Pme I and 10 µg of the liberated viral genome was transfected into a 60-mm dish of 116 cells expressing Cre recombinase [31]. One day after transfection, the transfected 116 cells were infected with the E1-deleted serotype 5 (Ad5) helper virus AdNG163 [10]. The packaging signal of AdNG163 is “floxed” or flanked by loxP sites for deletion during virus production in Cre-expressing cells. 48 hours later, crude lysates from this transfection/infection were amplified by serial coinfections of the crude lysate from the previous passage and AdNG163. Large-scale HD-Ad were produced by infection of 3 liters of 116 cells as previously described [31] and routinely produces HD-Ad preps with E1-E3-deleted helper virus contamination below 0.02% [31]. FG and HD-Ad virions were purified by CsCl banding and concentrations were determined by OD260 and real-time PCR. HD-Ad1, 2, and 6 vectors were also generated by infection with HD-Ad1, 2, or 6 floxed helper viruses Ad1LC8cCEVS-1, Ad2LC8cCARP [25], and Ad6LC8cCEVS-1, respectively. Ad1LC8cCEVS-1 and Ad6LC8cCEVS-1 were kindly provided by Carole Evelegh and Frank L. Graham (McMaster University).

### Animals

All animal experiments were carried out according to the provisions of the Animal Welfare Act, PHS Animal Welfare Policy, and the principles of the NIH Guide for the Care and Use of Laboratory Animals, and the policies and procedures of Mayo Clinic and University of Texas MD Anderson Cancer Center. Mice were purchased from Harlan Sprague Dawley Laboratories (Harlan, Indianapolis, IN) and maintained at Mayo Clinic. Eight adult male rhesus macaques (*Macaca mulatta*) of Indian origin between the ages of 8–17 years were obtained from and maintained in the specific pathogen-free breeding colony at the Michael Keeling Center for comparative medicine and research of The University of Texas MD Anderson Cancer Center, Bastrop TX. The animals were anesthetized during procedures to minimize discomfort.

### Mouse Immunizations

Mice were immunized intramuscularly (i.m.) and intravenously (i.v.). Mice immunized i.m. mice received 1×10^10^ vp/mouse in 50 µl. For immunization, 25 µl were injected into both quadriceps. Mice immunized i.v. received 1×10^10^ vp/mouse in 100 µl by tail vein injection. For serotype switching experiments, mice were boosted at 4, 9 and 15 wks after immunization. Groups of mice were immunized with homologous or heterologous HD-Env. Splenocytes and sera were harvested 4 wks post-immunization or at the final time-point for serotype switching and time-dependent studies.

### Luciferase Imaging of Mice

Molecular light imaging of luciferase *in vivo* was accomplished using a Lumazone imaging system (Roper Scientific). At 1 and 7 days post- injection, mice were anesthetized with Isoflurane, injected i.p. with d-luciferin at a concentration of 20 mg/ml in PBS in a volume of 200 µl and the mice were immediately placed into the Lumazone Imager and images were captured. All images were taken with a 10 minute exposure and 2×2 binning using no filters and no photo-multiplication. Data analysis was performed on each image using background subtracted mean intensities detected by the Lumazone Imaging Software at each time point and graphed using Prism Graphing Software.

### Enzyme Linked Immunosorbent Assay (ELISA)

To measure humoral immune responses to transgenes ELISAs were performed on mouse sera as previously described [33]. Briefly, Immulon 4 HBX plates (Thermo, Milford, MA) were coated with 100 µl of HIV-1 envelope (Env) protein, SF162 gp120 (NIH AIDS Reagent and Repository) or FireFly luciferase (Roche, Switzerland) at 1 µg/ml in PBS for 2 hours at room temperature (RT). The plates were blocked for 1 h with BSA at 2 mg/ml for 1 hour. Sera were diluted 1∶50 in PBS with BSA (1 mg/ml) and added to the plate for 1 h at RT. The plates were washed with 5 times PBS and 100 µl of Goat anti-mouse HRP conjugated antibody (Pierce, Rockford, IL) diluted 1∶2000 in PBS with BSA (1 mg/ml) was added to the plate for 1 h at RT. The plates were washed 5 times with PBS and 100 µl of 1 Step Ultra TMB-ELISA substrate (Pierce, Rockford, IL) was added for 1 h at RT. The reaction was stopped with 50 µl of 2 M sulfuric acid and analyzed at 450 nm using a Beckman Coulter DTX 880 Multimode Detector.

### Enzyme-Linked Immunospot (ELISpot) assay

To measure cellular responses to GFP and Env, splenocytes were incubated in the presence of peptides at a concentration of 5 µg/ml. GFP CTL responses were determined using the peptide HYLSTQSAL. The JRFL envelope CTL responses were determined using the HIV-1, subtype B, strain MN peptide RKRIHIGPGRAFYTT [34]. Anti-Ad5 cellular responses were determined using naïve splenocytes that were infected with wild-type Ad5 as antigen presenting cells (APC). Briefly, splenocytes were harvested from naïve BALB/c mice. The splenocytes were infected with wild-type Ad5 virus at 10,000 vp/cell for 1 hr at 37°C. The splenocytes were washed twice with incomplete DMEM and resuspended in complete DMEM containing 10% FBS and incubated overnight at 37°C. The infected splenocytes were then used as APCs in the ELISPOT assay. The spleens from individual mice were minced and then forced through a 40 µm Nylon cell strainer (BD Labware, Franklin Lakes, NJ). Single-cell suspensions of splenocytes were plated in 96-well polyvinylidene difluoride-backed plates (MultiScreen-IP, Millipore, Billerica, MA) coated with 50 µl of anti-mouse IFN-γ mAb AN18 (5 µg/ml; Mabtech, Stockholm, Sweden) overnight at 4°C. The plates were blocked with Hepes buffered complete RPMI medium at 37°C for 2 hr. Equal volumes (50 µl) of each peptide pool and splenocytes (10^7^ cells/ml) were added to the wells in duplicate. Plates were incubated overnight (14 to 16 hr) at 37°C with 5% CO_2_. After the plates were washed 6 times with PBS, 50 µl of 1∶1000-diluted biotinylated anti-mouse IFN-γ mAb (Mabtech, Stockholm, Sweden) was added to each well. Plates were incubated at RT for 2 hr and then washed 3 times with PBS. Fifty microliters of streptavidin-alkaline phosphatase conjugate (1∶1000 dilution; Mabtech, Stockholm, Sweden) were added to each well. After incubation at RT for 1 hr, the plates were washed 5 times with PBST. Finally, 100 µl of BCIP/NBT (Plus) alkaline phosphatase substrate (Moss, Pasadena, MD) were added to each well. The plates were incubated at RT for 10 min. After washing with water, plates were air-dried. Spots were counted using an automated ELISpot plate reader (Immunospot counting system, CTL Analyzers, Cleveland, OH) and expressed as spot-forming cells (SFC) per 10^6^ splenocytes.

### Tetramer Staining

Tetramers displaying the MHCI CTL epitope IGPGRAFYTT were obtained from the NIAID MHC Tetramer Core Facility. Mouse whole blood was collected in microtainer tubes containing K_2_EDTA (Becton Dickinson, Franklin Lakes, NJ). The RBCs were lysed using ACK Lysis buffer and washed twice with DPBS. Following lysis, 0.2 µg PE-labeled D^d^/P18 tetramer and FITC-labeled anti-mouse CD8α mAb (Ly-2, BD Pharmingen, Franklin Lakes, NJ) were used to stain P18-specific CD8^+^ T cells. The cells were washed in PBS containing 1% FBS and fixed in 0.5 ml PBS containing 1.5% paraformaldehyde. Samples were analyzed by two-color flow cytometry on a FACSCalibur (BD Biosciences, Mountain View, CA). Gated CD8^+^ T lymphocytes were examined for staining with the D^d^/P18 tetramer.

### Macaque Immunizations

Eight macaques from a previous study were used in these experiments. All eight animals received two immunizations of 10^11^ vp of Ad-EnvPeptide intranasally approximately 9 months prior to immunization with HD-Ad in this study. Ad-EnvPeptide expresses six conserved HIV-1 envelope peptides from a FG-Ad5 vector and [33]. These animals also received six synthetic env peptides adjuvanted with inactivated cholera toxin or with autologous dendritic cells during their prior immunizations. These synthetic peptides and Ad-EnvPeptide do not generate antibody-responses against HIV-1 envelope. In this study, these previously FG-Ad5-immunized macaques were immunized at days 0, 24, and 67 with 10^11^ vp of the indicated HD-Ads by i.m. injection. Serum samples were collected on days 24, 67, and 100 and 1/50 dilutions were analyzed for antibodies against envelope by ELISA as described above.

### Statistical Analyses

Data was evaluated using GraphPad Prism 4 software. Unpaired, two-tailed TTests and ANOVA with Bonferroni post test were used to determine statistical significance. P values≤0.05 were considered statistically significant.

## Results

### Comparison of *In vivo* Transduction by Replication-competent Ad (RC-Ad), FG-Ad, and HD-Ad Vectors

In gene therapy tests, HD-Ad vectors have been shown to be less immunogenic, have improved safety, and mediate extended expression of transgene products relative to FG vectors [19], [20], [21], [26]. This extended expression is thought to be due to the complete deletion of all Ad open reading frames (ORFs)(Fig. 1C) to reduce T cell responses against Ad antigens in transduced cells. In contrast, conventional FG-Ad vectors are deleted for only E1 and sometimes E3 gene products (Fig. 1B). While this removes E1 and E3 antigens and reduces expression of other Ad proteins, at least 17 ORFs are still present and some are expressed and can targeted by CTLs [15], [16], [17].

In order to determine the overall ability of the vector platforms to transduce cells in vivo we compared all three platforms, RC, FG and HD, using the Ad 5 serotype. RC-Ad was used as a positive control for this experiment since it is thought to produce stronger immune responses and toxicity due to replication and production of viral antigens than replication-defective vectors.

**Figure 2:**
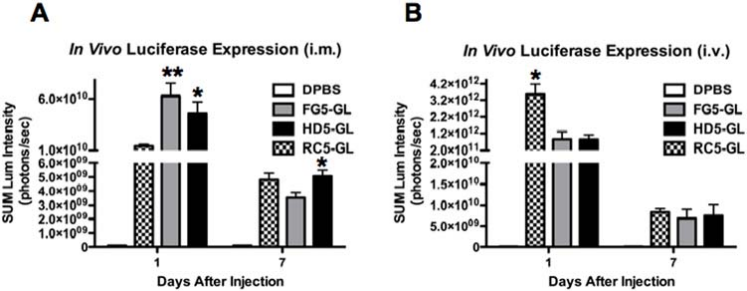
Luciferase imaging. *In vivo* transduction and expression produced by RC-Ad, FG-Ad, and HD-Ad expressing GFPLuc after i.v. (A) and i.m. (B) immunization. Groups of 5 mice were administered 10^10^ vp of the indicated vectors by the indicated routes. The animals were anesthetized, injected with luciferin, and imaged for luciferase activity at 1 and 7 days after injection. Data is shown for sum luciferase intensity for the muscles in i.m.-injected mice and for the liver in i.v.-injected mice. Data is the mean of luciferase activity from 5 mice for each group and error bars indicate standard error. The data were analyzed by one-way ANOVA (* = p<0.05 and ** = p<0.01).

RC5, FG5 and HD5 vectors expressing the GFPLuc (GL) transgene were injected i.m. and i.v. into BALB/c mice and the overall transgene expression was determined by luciferase imaging (Fig. 2). Luciferase expression levels after i.m. injection are shown in figure 2A. At day 1 after i.m. injection, both FG5-GL and HD5-GL had significantly higher luciferase expression as compared to RC5-GL (p = <0.01 and <0.05, respectively). Although all vectors had reduced luciferase expression levels at day 7, HD5-GL had significantly higher levels as compared to FG5-GL (p = <0.05) (Fig. 2A). Luciferase expression levels after i.v. injection are shown in figure 2B. Overall transgene expression was greater than 10-fold higher in i.v. injected mice as compared to i.m. injected mice. The most significant difference was seen in the RC5-GL injected mice where expression levels were more than 300 fold higher than i.m. expression levels and is most likely due to replication in the liver. Although reduced expression levels were observed for both FG5-GL and HD5-GL vectors after i.v. injection as compared to RC5-GL at day 1, there were no significant differences between the two replication-defective vectors (Fig. 2B). Expression levels for all vector platform were markedly reduced by day 7 and there were no significant differences between all vector platforms (Fig. 2B).

### Comparison of Transgene-directed T Cell Responses

**Figure 3:**
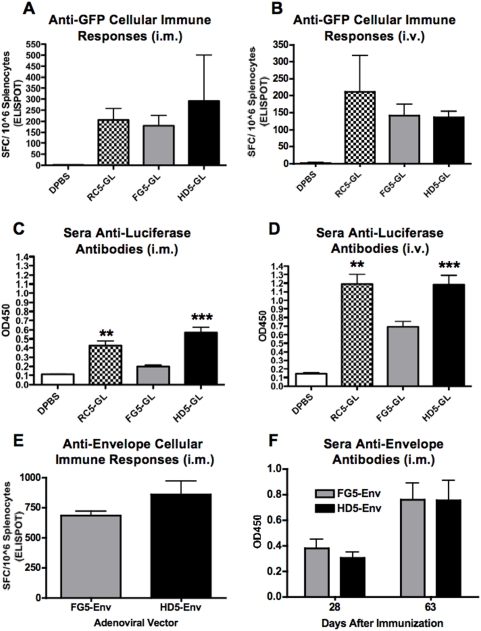
Humoral and cellular immune responses. Vector induced humoral and cellular immune responses against the transgene products were quantitated. Anti-GFP cellular immune responses induced by i.m. (A) and i.v. (B) injection are shown as IFN-γ spot forming cells (SFC) as measured by ELISPOT of splenocytes 4 weeks after immunization. Splenocytes were pulsed with H-2K^d^-restricted GFP peptide. Data is the mean from 5 mice for each group and error bars indicate standard error. Anti-luciferase antibody responses in sera from the i.m (C) and i.v. (D) immunized mice were collected 4 weeks after injection and were assayed by ELISA. Cellular and antibody immune responses against the HIV-1 envelope transgene are shown were quantitated after i.m. immunization with FG and HD vectors (E and F, respectively). Data is the mean ELISA OD450 from 5 mice for each group and error bars indicate standard error. The data were analyzed by unpaired two-tailed TTEST (** = p<0.01 and *** = p<0.001).

In order to determine if any one vector platform was better at inducing cellular immune responses, mice were immunized i.m. and i.v. with all three vectors expressing GFPLuc using the Ad5 serotype. The animals were sacrificed at 4 weeks post-immunization and their splenocytes were tested for T cell responses against GFP by ELISPOT (Fig. 3A and B). All vector platforms induced equivalent T cell responses against the GFPLuc transgene. There were no statistically significant differences in anti-GFP cellular immune responses after i.m. immunization with RC, FG or HD vectors (Fig. 3A). Similarly, i.v. immunization produced equivalent anti-GFP T cell responses by all vectors as evidenced by ELISPOT (Fig. 3B). We also compared the ability of FG5-Env and HD5-Env to induce anti-Env cellular immune responses (Fig. 3E). RC5-Env was not available for comparison. We found that both FG5 and HD5 platforms induced anti-Env cellular responses equally and there were no significant differences (Fig. 3E).

### Comparison of Transgene-directed Antibody Responses

Serum was drawn from the GFPLuc-immunized animals 4 weeks after immunization and was tested for antibodies against luciferase (Fig. 3C and D). Both RC5-GL and HD5-GL induced significantly higher levels of Anti-luciferase antibodies after i.m. injection than FG-Ad (p = <0.01 and <0.001, repectively) (Fig. 3C). Luciferase antibodies were approximately 2-fold higher by the i.v. route for all vectors. Similarly, i.v. injection of RC5-GL and HD5-GL also produced significantly higher levels of anti-luciferase antibodies than FG-Ad (p = <0.01 and <0.001, respectively) (Fig. 3D). Interestingly, HD-Ad produced antibody responses that were equal to or higher than those produced by RC-Ad by both routes. In contrast, FG-Ad antibody levels were approximately one half of the levels produced by HD-Ad consistent with previous studies [24]. These data indicate that HD-Ad vectors produce antibody levels comparable to replication-competent Ad and higher than FG-Ad. We also compared the ability of FG5-Env and HD5-Env to induce anti-Env antibody responses (Fig. 3F). We found that both FG5 and HD5 platforms induced anti-Env antibody responses equally well at both 4 wks and 9 wks post-immunization and there were no significant differences (Fig. 3F).

### Comparison of Vector Safety

**Figure 4:**
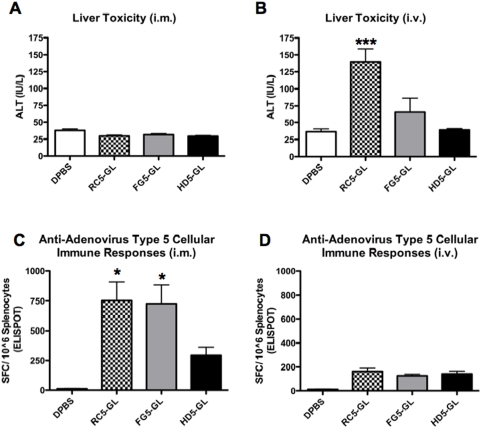
Toxicity and anti-vector cellular immune responses. Toxicity and the induction of anti-vector cellular immune responses were measured. Liver toxicity is expressed as a measured of ALT expression 48 hours after i.m. (A) and i.v. (B) injection. Anti-adenovirus cellular responses were measured 4 wks after i.m. (C) and i.v. (D) injections. Naïve BALB/c splenocytes infected with wild-type Ad5 were used as antigen presenting cells and were co-cultured with splenocyted from immunized mice. Anti-Ad5 cellular responses were measured by ELISPOT. Data represent groups of 5 mice and error bars indicate standard error. The data were analyzed by one-way ANOVA (* = p<0.05 and *** = p<0.001).

In mice, one side effect of Ad administration is liver damage after i.v. injection. To compare the toxicities of the three vectors, each was injected i.m. or i.v. into groups of 5 mice and liver enzyme ALT levels were measured in the blood 48 hours later (Fig. 4A and B). When ALT levels were tested after i.m. injection, no increases were observed with any of the vectors consistent with their sequestration from the blood and the liver (Fig. 4A). As expected, RC5-GL generated the highest ALT levels after i.v. injection that were significantly higher than FG5-GL and HD5-GL (p = <0.001). In contrast, HD5-GL produced only background levels of ALT consistent with previous results [23]. Although considered safer than RC-Ad, FG5-GL produced liver damage that was intermediate between RC5-GL and HD5-GL indicating that the first generation vector is safer than RC-Ad, but less safe than HD-Ad (Fig. 4B). This is consistent with previous data showing lower liver damage by HD-Ad than FG-Ad [23].

### Comparison of Ad5-directed T Cell Responses

ELISPOTs were also performed against Ad5 using splenocytes infected with wild-type Ad5 (Fig. 4C and D). Anti-Ad cellular immune responses were up to five-fold higher after i.m. injection (Fig. 4C) as compared to i.v. immunization (Fig. 4D). In this case, both the RC5-GL and FG5-GL vectors generated significantly higher Anti-Ad5 cellular responses (p = <0.05) as compared to the HD-Ad vector indicating that the absence of viral genes in the vector blunted the level of vector-directed T cell responses. In contrast Anti-Ad responses were lower by the i.v. route than the i.m. route. By the i.v. route all vectors produced relatively low responses and no significant differences with averages of 150 IFN-γ SFCs (Fig. 4D).

### Serotype-switching HD-Ad Vectors for HIV Vaccination

**Figure 5:**
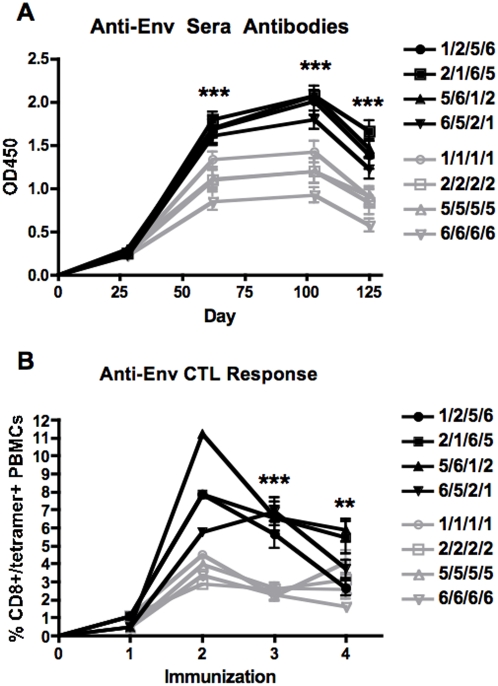
Anti-env humoral and cellular responses induced by HD-Ad serotypes. Groups of 20 BALB/c mice were immunized i.m. with 10^10^ vp of HD-Ad vectors expressing the HIV-1 JRFL gp140 env. A) Sera antibodies were assayed by ELISA 4 wks after priming, prior to boosting and 3 wks after final boosting. B) Tetramer staining was perfomed 4 4 ks after priming, prior to boosting and 3 wks after final boosting. The percent CD8+/tetramer+ cells was determined by flow cytometry. Data is the mean activity from 20 mice for each group for priming and 10 mice for each group boosted. Error bars indicate standard error and the data were analyzed by two-way ANOVA (** = p<0.01 and *** = p<0.001).

To test the utility of HD-Ad for serotype-switching, an HD5 vector expressing the HIV-1 JRFL envelope (Env) was packaged by Ad1, 2, 5, or 6 helper viruses and the resulting virions were used for i.m. immunization in mice (Fig. 5). Each HD-Ad produced similar anti-env antibodies when sera were assayed 4 weeks later by ELISA (Fig. 5A). When T cells in the blood were assayed by flow cytometry with env-specific MHC I tetramers, CD8+/tetramer+ cells were detected in all groups with HD1-Env and HD2-Env generating two-fold higher responses than HD5 and HD6 vectors after the first immunization (Fig. 5B).

**Figure 6:**
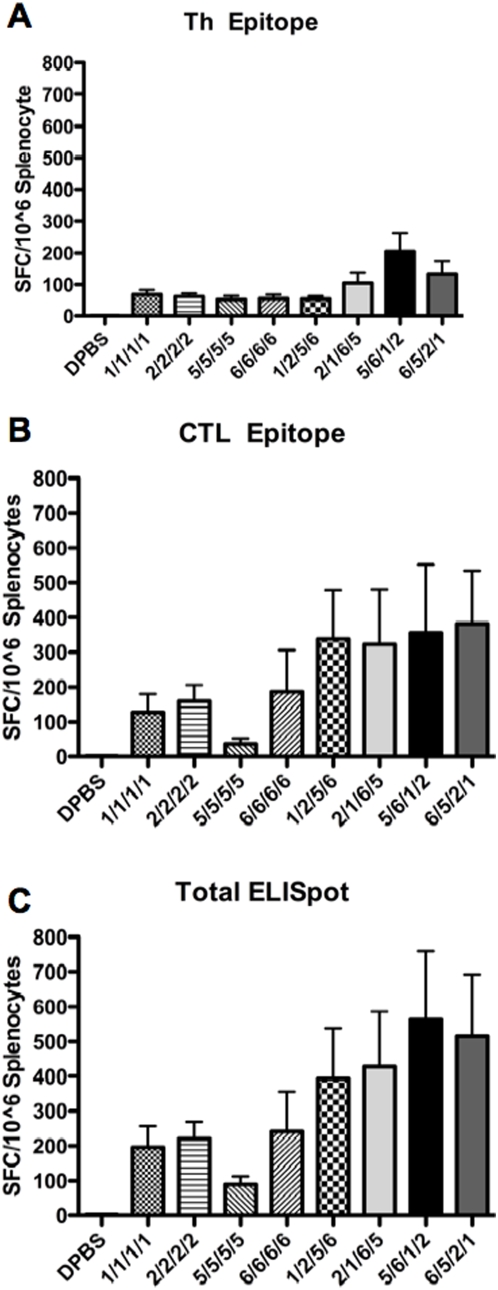
Effects of HD serotype switching on boosting anti-HIV T-helper and CTL responses. Mice were immunized i.m. with HD-Ad expressing HIV-1 gp140 Env. The mice were boosted with homologous or heterologous HD-Ad 3 times before ELISPOT assays were performed. Splenocytes from immunized mice were isolated and stimulated with T-helper (Th) epitope peptides (A) or a CTL epitope peptide (B). The total anti-Env cellular immune responses are also shown (C). Data is the mean activity from 10 mice for each group. Error bars indicate standard error and the data were analyzed by unpaired two-tailed TTEST.

To test serotype-switching of one HD-Ad vector backbone, the HD-Ad vectors were used for prime-boost immunizations. The groups of 20 mice that were primed with the four HD-Ad serotypes were each split into two groups of mice and they were boosted with either homologous HD-Ad or a heterologous HD-Ad serotype vector also expressing env. Four weeks after boosting, blood was collected and evaluated for humoral and cellular immune responses to HIV Env (Fig. 5A and B, respectively). All heterologous prime-boost regimens induced higher Anti-Env ELISA titers as compared to homologous prime-boosted mice (Fig. 5A). All mice boosted with heterologous HD-Env had significantly higher Anti-Env antibody titers at all post-boost time points (p = <0.001) (see Supplementary Table S1). Cellular responses assessed by MHC I tetramer staining were also higher in mice immunized with heterologous prime-boost regimens, as compared to homologous prime-boosting (Fig. 5B). Because the PBMCs were pooled for tetramer analysis after the second immunization, statistical analyses were not performed at that time point. There were no statistical differences between HD-Ad serotypes after the first immunization. However, analysis by two-way ANOVA after the third and fourth immunization showed statistically significant higher tetramer responses in mice boosted with heterologous HD-Ad with some p values<0.001 (Supplementary Table S2). After the fourth immunization, env antibodies declined in all groups, perhaps due to saturation of the responses or in response to regulatory cells. Third and fourth round immunization did not boost T cell responses in either group, however, tetramer levels remained statistically higher in all of the heterologous boosted mice after the third immunization and some of the heterologous boosted mice after the fourth immunization as compare to the homologous boosted mice (Fig. 5B). At the end of the study, the animals were sacrificed and their T cell responses were assayed from splenocytes by ELISPOT (Fig. 6). Stimulation of the cells with MHC II (Fig. 6A) or MHC I-restricted peptides (Fig. 6B) generated lower numbers of SFCs from the mice that were immunized only with one serotype of HD-Ad. Total SFCs were 2 to 4-fold higher in most of the groups immunized by serotype-switching with the HD-Ad vectors (Fig. 6C).

### Testing HD-Ad Serotype-switching in FG-Ad5-immune Macaques

A group of eight macaques from another study were available to test the HD-Ad vaccines. These macaques had previously been immunized with various formulations of six conserved HIV-1 env peptides (see Materials and Methods). Of interest to our work, these animals had also been immunized twice intranasally with FG-Ad5-EnvPeptide [33]. Since they had been exposed to Ad5 intranasally, these animals had the potential to mimic a natural Ad5 respiratory infection. In addition, these peptides generate T cell responses against HIV-1 envelope, but do not produce antibody responses against the protein [35], [36], [37], [38], [39]. Therefore, they could be used to evaluate anti-env antibody responses driven by HD-Ad vectors.

**Figure 7:**
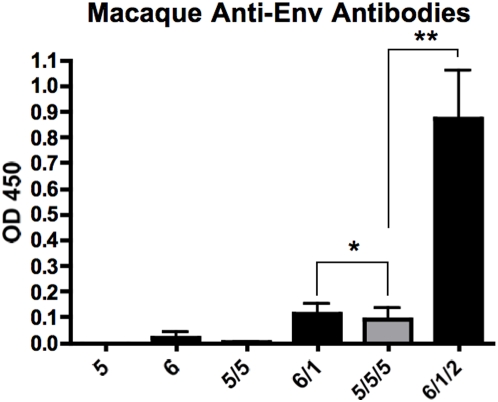
Evaluation of serotype switch in pre-immune macaques. Pre-immune macaques were immunized with 10^11^ vp of homologous or heterologous HD-Ad expressing HIV-1 gp140 Env. The macaques were boosted with 10^11^ vp of homologous or heterologous HD-Ad at days 24 and 67. Sera was collected on days 24, 67 and 100 and used to measure Anti-Env ELISA antibodies. Data is the mean of 4 macaques per group and error bars indicate standard error. Data was analyzed by one way ANOVA (* = p<0.05 and ** = p<0.01).

To test this, two groups of 4 macaques were immunized i.m. with 10^11^ vp of HD-Ad expressing HIV-1 env JRFL gp140 (HD-Env). On day 0, group one received HD5-Env and group two received HD6-Env by the i.m. route. On day 24, group one again received HD5-Env and group two received HD1-Env. On day 67, group one received HD5-Env for a third time whereas group two received HD12-Env. Serum samples were collected on days 24, 67, and 100 and evaluated by ELISA for anti-env antibodies (Fig. 7). FG-Ad5-immune animals that were immunized three times with HD5-Env generated only minimal antibody responses. In contrast, FG-Ad5 immune animals that were immunized with HD6-Env, HD1-Env, and HD2-Env generated detectable anti-env antibodies at each immunization with final antibody levels being 10-fold higher than in the HD-Ad5 group (p<0.01). These data demonstrate the breadth of a HD-Env vector packaged by multiple serotype helper viruses to evade pre-existing or vector induced immune responses.

## Discussion

This study was directed at determining if HD-Ad vectors would have utility as gene-based vaccine platforms. To evaluate both vaccine potential and vector side effects, we compared HD5-Ad to replication-defective FG5-Ad and replication-competent RC5-Ad. These data demonstrate that HD-Ad and RC-Ad both generate stronger immune responses than FG-Ad. In contrast, FG-Ad and RC-Ad both generated higher anti-Ad T cell responses and liver damage indicating that HD-Ad has a better safety profile than either of these vectors. These data indicate that HD-Ad vaccines generate strong immune responses against gene-based antigens, but have reduced side effects. This data is consistent with previous gene expression and safety work comparing HD-Ad and FG-Ad for gene therapy applications [10], [26]. It is also consistent with previous work comparing the ability of FG-Ad and HD-Ad expressing β-galactosidase to generate immune responses [24]. In this case, HD-Ad vector also generated stronger T cell and antibody responses than FG-Ad [24].

In light of the HIV-1 human vaccine STEP trial results, HD-Ad vectors may have the an advantage in not expressing any Ad antigens from transduced cells. As shown in Figure 1, conventional E1/E3-deleted FG-Ad vectors still carry 17 potential vector antigen ORFs whereas HD-Ad has zero. While E1 and E3 deletion renders FG-Ad vectors largely (but not completely) replication-defective, this still allows Ad proteins to be expressed in a leaky fashion [13]. It appears that this leaky expression in transduced cells is what stimulates new T cell responses and recall T cell responses against the vector which destroy transduced cells. If Ad-specific T cell responses are involved in increasing HIV-1 acquisition in the STEP trial, then it is possible that an HD-Ad vector would reduce this side effect. This is suggested by the lower anti-Ad T cell responses generated by HD-Ad as compared to both FG-Ad and RC-Ad.

While HD-Ad did have lower anti-Ad T cell responses, they were not zero. This is likely due to T cell responses due to the delivery of Ad antigens from the incoming HD-Ad virions. One approach to mitigate T cell and antibody responses versus Ad due to protein delivered in the virion would be coating the virus with polymers like polyethylene glycol (PEG). Indeed, PEGylated HD-Ad vectors appear as robust as unmodified vectors [23], [40]. This effect of carried protein antigen can also largely be obviated by using HD-Ad vectors from infrequently observed Ad serotypes and performing serotype switching [21], [25], [27], [28], [29]. Towards this end, we show that serotype switching of HD-Ad vectors is simple by using alternate serotype helper-viruses from the same Ad subgroup. We show that one HD-Ad genome can be packaged by four different helpers and that each of these generates robust HIV-directed immune responses. Serotype switching allowed for multiple rounds of boosting that increased anti-Env immune responses significantly as compared to homologous boosting. In general, the anti-Env immune responses plateaued after the third immunization. This may be due to a down regulation of anti-Env responses by regulatory cells or by saturation of the transgene product by circulating anti-Env antibodies. While these serotypes were convenient to test proof of principle as they were existing vectors, Ad1, 2, and 5 serotypes are not optimal, since pre-existing immunity in humans range from 27 to 50% for these viruses. In contrast, pre-existing immunity to Ad6 may be as low as 3% [14], which may make HD-Ad6 of interest in a DNA prime- HD-Ad6 boost or in combination with HD-Ads produced from less prevalent serotypes.

In summary, HD-Ad vectors produce immune responses equal to or better than FG and RC Ad vaccines that carry viral ORFs. HD-Ad vaccines produce lower side effects and vector-directed T cell responses likely due to the absence of these viral genes in the vector. HD-Ad serotype-switching proved effective at generating stronger immune responses against HIV-1 envelope in both mice and FG-Ad5-immune macaques. Based on this, HD-Ad vectors from low seroprevalence adenoviruses may have utility as vaccines for HIV and other pathogens.

## Supporting Information

Table S1 (0.06 MB PDF)

Table S2 (0.08 MB PDF)
