# Supplementary material for: Comparison of Replication-Competent, First Generation, and Helper-Dependent Adenoviral Vaccines
Source: PLoS One. 2009 Mar 31;4(3):e5059. doi: 10.1371/journal.pone.0005059 (PMC2659436; doi:10.1371/journal.pone.0005059)
Supplement: Table S2 — (0.08 MB PDF) [file pone.0005059.s002.pdf]

Supplemental Table 2.

Two-way analysis of variance for anti-Env CTL responses induced by immunization with HD-Env vectors

Prime

	1/2/5/6	2/1/6/5	5/6/1/2	6/5/2/1	1/1/1/1	2/2/2/2	5/5/5/5	6/6/6/6
**1/2/5/6**	-							
**2/1/6/5**	> 0.05	-						
**5/6/1/2**	> 0.05	> 0.05	-					
**6/5/2/1**	> 0.05	> 0.05	> 0.05	-				
**1/1/1/1**	> 0.05	> 0.05	> 0.05	> 0.05	-			
**2/2/2/2**	> 0.05	> 0.05	> 0.05	> 0.05	> 0.05	-		
**5/5/5/5**	> 0.05	> 0.05	> 0.05	> 0.05	> 0.05	> 0.05	-	
**6/6/6/6**	> 0.05	> 0.05	> 0.05	> 0.05	> 0.05	> 0.05	> 0.05	-

Boost 1

	1/2/5/6	2/1/6/5	5/6/1/2	6/5/2/1	1/1/1/1	2/2/2/2	5/5/5/5	6/6/6/6
**1/2/5/6**	-							
**2/1/6/5**	ND	-						
**5/6/1/2**	ND	ND	-					
**6/5/2/1**	ND	ND	ND	-				
**1/1/1/1**	ND	ND	ND	ND	-			
**2/2/2/2**	ND	ND	ND	ND	ND	-		
**5/5/5/5**	ND	ND	ND	ND	ND	ND	-	
**6/6/6/6**	ND	ND	ND	ND	ND	ND	ND	-

Boost 2

	1/2/5/6	2/1/6/5	5/6/1/2	6/5/2/1	1/1/1/1	2/2/2/2	5/5/5/5	6/6/6/6
**1/2/5/6**	-							
**2/1/6/5**	> 0.05	-						
**5/6/1/2**	> 0.05	> 0.05	-					
**6/5/2/1**	> 0.05	> 0.05	> 0.05	-				
**1/1/1/1**	< 0.001	< 0.001	< 0.001	< 0.001	-			
**2/2/2/2**	< 0.001	< 0.001	< 0.001	< 0.001	> 0.05	-		
**5/5/5/5**	< 0.001	< 0.001	< 0.001	< 0.001	> 0.05	> 0.05	-	
**6/6/6/6**	< 0.001	< 0.001	< 0.001	< 0.001	> 0.05	> 0.05	> 0.05	-

Boost 3

	1/2/5/6	2/1/6/5	5/6/1/2	6/5/2/1	1/1/1/1	2/2/2/2	5/5/5/5	6/6/6/6
**1/2/5/6**	-							
**2/1/6/5**	< 0.001	-						
**5/6/1/2**	< 0.001	> 0.05	-					
**6/5/2/1**	> 0.05	< 0.05	< 0.001	-				
**1/1/1/1**	> 0.05	> 0.05	< 0.01	> 0.05	-			
**2/2/2/2**	< 0.001	< 0.001	< 0.001	> 0.05	> 0.05	-		
**5/5/5/5**	> 0.05	< 0.001	< 0.001	> 0.05	< 0.05	> 0.05	-	
**6/6/6/6**	> 0.05	< 0.001	< 0.001	< 0.01	< 0.001	> 0.05	> 0.05	-

ND = not determined
